# A “Misfit” Theory of Spontaneous Conscious Odor Perception (MITSCOP): reflections on the role and function of odor memory in everyday life

**DOI:** 10.3389/fpsyg.2014.00064

**Published:** 2014-02-11

**Authors:** Egon P. Köster, Per Møller, Jozina Mojet

**Affiliations:** ^1^Psychological Laboratory, Helmholtz Institute, Utrecht UniversityUtrecht, Netherlands; ^2^Department of Food Science, University of CopenhagenFrederiksberg, Denmark; ^3^Wageningen – UR, Food and Biobased ResearchWageningen, Netherlands

**Keywords:** incidental learning, implicit memory, olfactory perception, ecological validity

## Abstract

Our senses have developed as an answer to the world we live in (Gibson, 1966) and so have the forms of memory that accompany them. All senses serve different purposes and do so in different ways. In vision, where orientation and object recognition are important, memory is strongly linked to identification. In olfaction, the guardian of vital functions such as breathing and food ingestion, perhaps the most important (and least noticed and researched) role of odor memory is to help us not to notice the well-known odors or flavors in our everyday surroundings, but to react immediately to the unexpected ones. At the same time it provides us with a feeling of safety when our expectancies are met. All this happens without any smelling intention or conscious knowledge of our expectations. Identification by odor naming is not involved in this and people are notoriously bad at it. Odors are usually best identified via the episodic memory of the situation in which they once occurred. Spontaneous conscious odor perception normally only occurs in situations where attention is demanded, either because the inhaled air or the food smell is particularly good or particularly bad and people search for its source or because people want to actively enjoy the healthiness and pleasantness of their surroundings or food. Odor memory is concerned with novelty detection rather than with recollection of odors. In this paper, these points are illustrated with experimental results and their consequences for doing ecologically valid odor memory research are drawn. Furthermore, suggestions for ecologically valid research on everyday odor memory and some illustrative examples are given.

## INTRODUCTION

According to Gibson (1966, 1979) our senses have developed as an answer to the world we live in and their diversity can be seen as the reaction to the different challenges our world poses. Thus, he describes the many senses involved in movement and kinesthesis (from skin pressure to joint angle sensitivity and vestibular orientation) as an answer to gravity. The intricate interplay of these sensory impressions remains implicit and escapes our conscious attention, making sure that we are never in doubt about our relative position with regard to the earth. The memory for our movements in relation to the weight of objects permits us to fulfill small wonders like making three pointers in basketball. Odor perception and odor memory are also most of the time implicit, but for a different reason. As Gibson indicated, smelling is an accompaniment of breathing, which is a vital function in all animals. As such it is sensitive to volatile “foreign substances” in the normally constant system of pure air that remains odorless. Here Gibson forgot to mention that the sense of smell is also watching over the foods we ingest by the retro-nasal stimulation occurring during eating. He only pays attention to the orthonasal stimulation and its function in food, mate finding, and in relation to prey/predator behavior. In doing so he adds to the conviction that identifying the odor source is the primary objective of olfaction. Before discussing odor perception and odor memory in more detail, it is perhaps useful to clarify the meaning of some of the terms used. Implicit perception and implicit memory refer too our unawareness of either perceiving something or having a memory of it. In everyday life our explicit and conscious perception and memory cover only a small part of what goes on. Memory is to a large part based on incidental learning that takes place without any intention to memorize and our memory is filled with knowledge that we use without special conscious attention. Olfactory memory is usually strongly, but implicitly linked to emotion and hedonic appreciation of our surroundings rather than to explicit odor source recognition. Thus, although it is true that odors may result in attraction or repellence and may have strong emotional and behavioral effects (Jacob and McClintock, 2000; Lundstrom et al., 2003; Chebat and Michon, 2003; Holland et al., 2005), it is doubtful that at least in human olfaction explicit or even implicit identification of the odor source necessarily plays a role. In fact, most well-known odors are not even consciously remarked and just provide a sense of safety. Only the odors that do not fit our memory based expectations, either because they deviate from the normal odor in that situation or by being particularly “good” or “bad,” are spontaneously and consciously remarked in normal everyday life. All expected odors are usually not. Each room in our house and even each room corner smells differently but we do not notice the hundreds of odors in our daily surroundings (Keller, 2011). Of course it is possible to actively smell them by sniffing, but in contrast to most animals, humans do seldom use active smelling, since due to their erect posture, they are primarily oriented by vision and audition. If they do smell actively, it is usually to verify the safety of the surroundings or to enjoy sensual pleasures. Thus, olfactory memory seems to play a very special role in life: it helps people “not to notice” known odors, but to react to all unexpected ones. One does not smell the odors in one’s own house, but notices the odors in the houses of friends. This might mean that, at least in humans, the implicit memory of odor perception could be more related to its passive function as a warning system for the breathing or ingestion of possibly dangerous odors or foods (see the section on incidentally learned food memory below), than to the active behavior of food and mate search that Gibson described. This passivity may perhaps also explain why odor evoked memories are more emotional than memories evoked by visual or verbal stimuli (Herz and Schooler, 2002; Herz, 2004). Only in special cases, when the odors are new or do not fit the situation (Herz, 1997) or when the situation is new or so exceptional that it puts all our senses on alert, will we note the odors, whereas under normal circumstances we do not. Nordin et al. (1995) showed that 77% of healthy elderly remain unaware of the fact that they have severe losses of olfactory sensitivity (even up to complete anosmia). White and Kurtz (2003) confirmed that elderly have little metacognitive awareness of their olfactory deficiencies and that this lack of awareness might be due to the slow disappearance of their sensitivity similarly to what occurs sometimes in loss of hearing. They also showed that metacognitive awareness of odor perception is also weak in young people who show a tendency to underestimate their olfactory capabilities. This seems to indicate that conscious odor perception is probably never such an important part of life as in hearing or vision where our communication with others and our environment depend on it. Complete loss of hearing or blindness would seldom go unremarked, but complete anosmia is often unnoticed. Along similar lines it can be explained why the bump in the autobiographical memory curve (i.e., the period of one’s lifetime to which most memories go back) evoked by odors lies much earlier (before 10 years) than that for visually or verbally evoked memories [between 15 and 25 years; (Chu and Downes, 2000, 2002; Willander and Larsson, 2006, 2007, 2008)]. Once known, odors are simply not very easily remarked anymore in later phases of life. As a result the first impressions with them are not replaced by later events involving them. Furthermore, since most authors used only odors that were known by most of their subjects already as a child and were thereafter seldom consciously experienced, they reduced the chances of association of these odors with later events. Unfortunately, none of the authors did specify the results for the odors they used. Otherwise it might have been possible to date their first contacts with them. According to the theory developed below, the chances to be linked to an autobiographical memory at a later age are slimmer for odors that were already perceived regularly (or even occasionally) in childhood. In the Proust (Proust, 1922/1960) phenomenon it is the rather unique combination of the madeleine and linden tea that, when the same combination arrives many years later unexpectedly in a different situation, evokes the memory of the Sunday mornings before mass in his aunts bedroom. Ordinary daily odors that are encountered in many different situations could not do this. Therefore keeping track of the frequency of occurrence of the individual odors and of the moments of first encounter with them in the life of individual subjects seems important in autobiographical research.

Another indication that olfaction does not seem to be interested in known odors is the fact that olfaction is a sense with complete adaptation. It means that the sensitivity for sustained monotonous stimulation is completely lost after a few minutes and that recovery from adaptation after cessation of the stimulus is slow. Nevertheless the sensitivity to new other odors remains largely intact (Köster, 1971; Köster and DeWijk, 1991). Moreover, complete adaptation seems to indicate that permanent awareness of experienced odors is not important and that it may even be harmful in as far as it might make us less vigilant and less attentive to the arrival of new and potentially dangerous odors in the very complex olfactory environment we constantly live in. In senses that play an active role in spatial orientation and movement such as vision and audition complete adaptation does not occur.

On the basis of the foregoing, we would like to formulate what we could call “the misfit theory of spontaneous conscious odor perception” (MITSCOP), a form of “perception by exception” guided by olfactory memories via the expectations about the odors in the situation. It plays, next to more semantic forms of explicit memory, a large role in incidental learning and implicit odor memory and is based on the following principles.

• In everyday life almost all odors are incidentally and unconsciously associated to the situation in which they occur and are stored as implicit expectations about their occurrence and not as precise recollections of the odor itself.
• An incidentally learned odor will not be spontaneously perceived consciously if it fits our implicit expectations in the situation, but if there is a misfit it will.

• Misfits may occur in different forms:
a. A novel or changed odor will be presented in the same situation
b. The original odor will be presented in a new situation.
c. The originally encountered odor or situation may acquire a new emotional value due to state dependent factors in the perceiver (hunger, emotional shock, extreme odor intensity perception, etc.)

• Explicit odor perception and memory are important only in a few instances of normal life (gas detection, cooking, etc.), but are of great significance in the work of odor and flavor experts. With respect to the normal role and function of odors in human everyday life, the ecological validity of explicit intentional odor perception and memory experiments involving the identification of odors as such, might be questioned.

In this paper, we will provide further evidence for such a view and we will discuss the existing literature on odor perception and memory research critically. Finally, we will formulate criteria for ecologically valid odor perception and memory research and we will try to indicate ways in which these criteria can be met. MITSCOP is proposed as a more parsimonious explanation of the fact that conscious olfaction is rare than the idea of a “constant state of olfactory change blindness” proposed by Sela and Sobel (2010) which can’t explain many of the phenomena discussed here. Their theory is based on the idea that sniffing is the only way in which odors become effective. Thus, it seems to exclude retronasal food perception and the many instances where subliminal odors influence behavior unconsciously.

## CONSCIOUS ATTENTION TO ODORS

Once an odor has been perceived for the first time in a certain situation we tend to pay no more attention to it in that situation, probably because it does not provide a threat and its implicit perception results in feelings of well-being and safety without conscious perception of the odor itself. This idea is one of the corner stones of MITSCOP. Although it may seem that this is just an instance of a very general attentional theory and not specific for olfaction, it should be pointed out that the role of familiarity and novelty detection seems to be different in olfaction and in vision. People are extremely sensitive to off-odors and off flavors (Nijssen, 1991) in very complex odor mixtures, but easily overlook changes in the visual surroundings and spend a long time finding the 10 differences in two-picture-puzzles or to locate Wally in “Where is Wally?” pictures. Conscious attention in odor perception and its necessity for effectiveness in present or later behavior has also been a subject of discussion. Herz (1997) insisted on drawing people’s attention to the odor during the encoding of her context-dependent memory tasks, whereas others (Kirk-Smith et al., 1983; Degel et al., 2001; Holland et al., 2005; Li et al., 2007; Zucco et al., 2009; Gaillet et al., 2013) carefully avoided drawing attention to the presence of odor in their incidental learning sessions. These latter authors clearly showed that conscious odor awareness is not a prerequisite for its effectiveness in behavioral modulation. Along another line, even the possibility to selectively direct one’s attention to olfaction has been doubted on the basis of the fact that olfactory information bypasses the thalamus, but Spence et al. (2000, 2001) have clearly established the possibility of modulating behavioral responses by selective attention to odors. The relationship between attention and olfactory consciousness was also extensively discussed in a review article by Keller (2011). In line with MITSCOP, he points out that “with almost every breath we inhale air containing odors at relatively high concentrations; yet olfactory experiences are very rare.” Furthermore, he points out that the involuntary increase in attention to odors which women may experience during pregnancy without change of olfactory acuity (Doty and Cameron, 2009) is probably an adaptive response to the fetuses’ special sensitivity to poison. There are also large differences in attention to olfaction among non-pregnant individuals. Nevertheless, it is true that in everyday life almost all people pay little conscious attention to odors and it remains surprising how little research has been done on the effects of unattended and unconscious odor perception.

## INCIDENTALLY LEARNED MEMORY FOR FOODS

**FIGURE 1:**
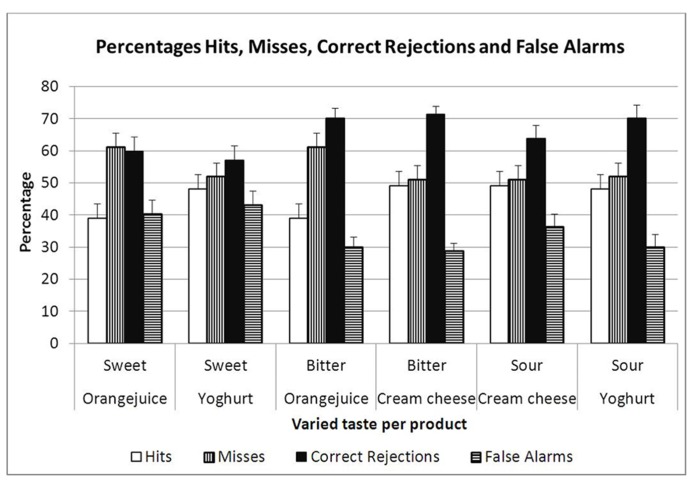
**Percentages of the four response types in each of six taste memory tests by Köster et al. (2004)**.

Perhaps the most extensive evidence for the misfit theory comes from food memory. The results of a number of different experiments (Morin-Audebrand et al., 2012) showed that memory for incidentally learned food properties (texture, flavor, taste) was based on detection of change rather than on recollection of the previous experience with the food. All experiments were based on a paradigm developed by Mojet and Köster (2002, 2005), in which people were exposed incidentally to foods and/or drinks during another experiment or a quasi-accidental meal without any reference to a memory task and were later unexpectedly asked to recognize these foods amidst distractors consisting in slight variations of that food which still had the same basic flavor, but in which one of the components (e.g., the sweetness, the fattiness, or the flavor, etc.) had been changed by a small, just detectable, amount. In these experiments the participants could not indicate the original food better than by chance, but they could detect very clearly that the distractors were not the ones they had had before. In other words they noted the misfits readily, but could not identify the earlier perceived food itself (see **Figure 1**).

To counter the idea that these results were based on a response bias on the part of the participants that favors the correct rejection of the variants and diminishes the hit rate, the certainty of the respondents in uttering their responses was also measured in most experiments. It showed that the participants were significantly more certain of their correct rejections of the variants than of any of the other three possible responses (Hits and Misses: saying yes or no to the earlier experienced one; False alarms: saying yes to a variant). Support for the fact that novelty detection prevails over recollection comes also from the work of Jehl et al. (1995), who showed that familiarization with odors did not affect the hit rate for these odors in a memory experiment, but significantly improved correct rejection of the distractors as shown in reduced false alarm rates. A similar support for novelty detection dominance was obtained in the interference experiments of Zucco (2003), who found that odor memory (in contrast to visual and auditory memory) was not affected by interference. In the discussion Zucco remarks “The assumption that people lack a conscious representation for odors could successfully explain any of these effects.” On the basis of recent incidental learning and recognition experiments (see below), the present authors take the more radical viewpoint that correct rejection of the distractors on the basis of their novelty in the experimental situation suffices to explain the results and that characteristics of the olfactory engram do not come into play in recognition at all. In other words, what is not there (the engram or recollection) cannot be interfered with or forgotten, but novelty (of distractors) is always functional. This might also explain the longevity of odor memory in recognition experiments (e.g., Engen and Ross, 1973) and why many authors fail to find serial position effects in odor memory (see overview Miles and Hodder, 2005). In most memory tests (visual as well as olfactory) the authors have used a two alternative forced choice test to measure the memory performance. Unfortunately this made it impossible to know whether the memory was based on recollection of the earlier experienced stimulus or on rejection of the distractor as being novel (see also the criticisms on the use of Signal Detection Theory in memory research discussed below). Thus, odors in the laboratory may not be remembered as such, but merely become linked to the experimental situation. When the situation is repeated only the distractors will be detected by their novelty in that situation and there is no need for recollection or re-activation of the engram characteristics of the earlier experienced stimuli. Indeed, it has been shown that incidentally presented odors are not even better remembered than by chance guessing, unless they are associated with a name or with an emotional event, but the new distractors are correctly rejected with great certainty and account for the memory performance (see below Degel et al., 2001; Møller et al., 2004, 2007; Morin-Audebrand et al., 2012).

Not having a specific memory of the odor characteristics is indeed perhaps also the best way to prevent extinction or loss under counter-conditioning as in the experiments of Stevenson (2001a,b,c). Again, what is not functional or is not even present can’t be lost or affected by new information. In vision and audition, where conscious representation is possible and recollection seems to prevail, interference occurs probably because the representations of the remembered and the new stimuli compete at the same level (Zucco, 2003). In vision there is also evidence of a dissociation between familiarity based and content related memories (Brown and Aggleton, 2001; Daselaar et al., 2006a,b,c), but since there are only few data on truly incidentally learned visual memory, it is not clear whether “feelings of not-knowing” play the same role as in olfaction. Novel events and the neural mechanisms for detecting and remembering them have also been discussed by Ranganath and Rainer (2003) distinguishing stimulus novelty and contextual novelty.

The findings in olfaction and eating behavior were interpreted (Köster, 2005) as indications that whereas identification of a possible danger source is important in vision where it may help to choose adequate action (hiding, aggression, submission, etc.), it is not important in olfaction, where only one possible reaction (holding one’s breath and fleeing, or spitting out in the case of food) is possible and time allowed for adequate reaction is short, because the stimulus is already at or in the body. Novelty and change detection might therefore have priority over identification. We are not only bad at odor identification, knowing the name of an odor may also make it lose its intimate connections to the situations in which it was first perceived, as the results of Willander and Larsson (2007), studying autobiographical memories, suggest. They compared memories evoked by respectively odors alone, odor names alone, and odors with their names and found that the high percentage of early autobiographical memories that came in the odor alone condition was very significantly reduced if the name was given with the odor. This suggests that “objectifying” the odor by naming it, makes it lose the emotional bond with specific life situations, which is so typical for the effects of odors in everyday life.

A further argument for MITSCOP was found in an extensive same-different judgment experiment with odors (Møller et al., 2012) showing that, contrary to same–different experiments carried out under comparable circumstances in vision where same detection is a bit faster than difference detection (Farell, 1985; Luce, 1986; Posner, 1986), in olfaction detecting a difference between two odors (a misfit) is much faster than detecting sameness. This strongly suggests that identification is important in vision but not in olfaction, where fast change detection is more important.

## IMPLICIT MEMORY FOR INCIDENTALLY LEARNED ODOR-PLACE ASSOCIATIONS

The most convincing demonstration that odor identification, being the most outspoken form of explicit awareness, is not a necessary prerequisite in odor memory comes from experiments demonstrating the memory relationship between odors and the places where they were present without being consciously noticed (Degel and Köster, 1998, 1999; Degel et al., 2001; Köster et al., 2002). It was shown that people who had been unknowingly exposed to very slight and consciously unnoticed odors in rooms in which they performed a psychological test, would later, in a seemingly unrelated experiment on room odor selection indicate the exposure odor as fitting the room much better than people who had not been exposed to odor in that room, but only when they could not identify the odor by name. If they did know the name of the odor the situational spell was broken and they reacted in the same way as the people who had not been exposed to odor in the room or had never been in it (see **Table 1**).

**Table 1:** Ratings of fit of the odors to the rooms by non-identifiers, identifiers, and non-exposed subjects (Degel etal., 2001).

Room	Room A	Room A	Room B	Room B	Total
Odor	Lavender	Orange	Lavender	Orange	
Non-identifiers	1.49^a^	0.83^a^	1.68^a^	1.31^a^	1.33^a^
Identifiers	0.61^b^	0.44^b^	0.89^b^	0.80^b^	0.69^b^
Non-exposed	0.62^b^	0.48^b^	0.76^b^	0.99^b^	0.73^b^

*Ratings with different letters in the columns are significantly different (*P* < 0.05).*

These results clearly show that objectifying odors by identifying and naming them makes them lose their probably most important function of secretly connecting us via memory to places and situations in our life that have emotional meaning. Others (Li et al., 2007) have also illustrated loss of function by conscious awareness of the odors. They showed that odors only had emotional effects on the judgment of faces when they were not consciously perceived. Furthermore it is well-known in the perfume industry that many ingredients (e.g., musk, civet) lose their effectiveness in a mixture at concentrations where they begin to be perceived (Köster and Degel, 2000). Such results also show us that we may be mistaken if we see odor identification as the penultimate goal of odor memory and they may help us understand the importance of the silent implicitness of odor memory in making us at home in our world. Odors are not meant to be objectified and identified and therefore we are so bad at it.

## TRADITIONAL ODOR MEMORY RESEARCH: FLAWS AND VIRTUES

If our misfit theory is right, most odor memory remains implicit using its “conscious perception effacing” function to make us feel well and safe by not noticing the expected. Therefore, one may ask why most odor memory research has been directed at explicit recognition and identification of odors that usually were learned in objectified form during explicit learning sessions. For even if no explicit memorizing demand is made, a laboratory session in which odors are presented as separate items in bottles (or by an olfactometer), is far removed from the incidental learning situations in normal life, where odor often is an ephemeral epiphenomenon of an otherwise attention demanding situation. In most laboratory experiments odors are treated as things independent of any situation at learning and, in analogy to memory for visual objects, memory for them is tested by asking for their recognition via recollection and identification amidst completely unrelated other odor items. Such an approach is not only ecologically invalid, but it differs also fundamentally from the methods for studying food memory described above. Nevertheless, it may provide insights in the working of odor perception and memory under such abnormal conditions, compared to those in other sensory modalities tested under the same conditions and help to elucidate differences between people in their odor sensitivity, discrimination and memory due to gender and age. It has been shown for instance that the time-curves of memory and forgetting for thus presented single odors resembles that of non-identifiable and unstructured visual shapes (Lawless, 1978) and differs from those found for identifiable visual pictures or words (Engen and Ross, 1973). In an experiment associating odors with two different pictures, Lawless and Engen (1977) also found that the first association was better remembered than the second one. They interpreted this finding as an indication of strong proactive inhibition. At the same time all these findings fit well in the misfit theory and the unimportance of odor identification.

Research with itemized single odors has also clarified important differences between implicitly and explicitly learned odor memory. In an experiment with very uncommon odors, chosen to avoid the possible influence of verbal memory, groups of elderly and young people were either incidentally exposed to the odors and judged them on pleasantness or were exposed under the instruction to remember them in view of a later test (Møller et al., 2004). It could be shown that the incidentally learned odor memory of the elderly was at least as good as and even slightly better than that of the young subjects, but that the young outdid the elderly significantly in the intentional learning condition. This result was later confirmed in an ecologically more appropriate experiment with soups and more natural incidental learning conditions (Møller et al., 2007). Thus, it can be seen that the unnatural conditions in the laboratory may be very informative, but that it is nevertheless good to verify their external validity by more ecologically based means. Perhaps the worst mistakes are based on the idea that explicit odor perception and odor memory are the normal ways of dealing with odors. Especially in the learning phase it is necessary to arrange things in a normal way without attracting extra attention to the odor and without any reference to memory. Thus, it might not be a good idea to ask people how often in their life they have encountered certain odors as the learning phase in an implicit odor memory experiment using repetition priming as Olsson (1999) and Olsson and Cain (2003) did. It invokes the thought of memory even if it does not contain a direct hint that the odors should be remembered. On the other hand, Olsson (1999) used a very good method trying to avoid the possible influence of semantic memory on the results in the later testing of the memory for the odor. Instead of asking people to recognize the previously presented and non-presented controls while measuring their reaction times, he familiarized the subjects with a special comparison stimulus and asked in the final test whether the presented stimulus was the comparison stimulus or not. He then compared the reaction times to the “no” responses given to the earlier primed stimuli and non-primed control stimuli. Under these conditions no overall priming effect was found, but further analysis of the data after the participants had also performed an identification test, showed that primed identifiable odor stimuli did significantly worse than identifiable control odors, whereas with unidentifiable odors the reverse was true, the primed ones showing shorter reaction times. This is in line with the data of Degel et al. (2001) on the effects of odor identification in incidentally learned memory for room odors (see **Table 1** above).

As indicated, it is often difficult to separate veridical odor memory (i.e., memory for the smell itself) from the semantic memory for the o*dor’s* name. Unfortunately, the overwhelming majority of odor memory studies falls prey to this confound (see Larsson, 1997). Investigations of veridical odor memory should not provide subjects with the possibility of remembering an odor by some verbal label. This can be avoided by using targets and distractors which belong to the same odor-category and which subjects would label in the same way, while still being able to perceptually discriminate between them. The methods used in the studies of food memory mentioned above (Morin-Audebrand et al., 2012) can easily be applied to other odor memory research. Another way to minimize the use of verbal labels is to apply stimuli which subjects do not have names for. An example of this is provided by Møller et al. (2004). Those who are fascinated by the question why it is so difficult to identify odors by name (Cain, 1982; Cain et al., 1998) or those who think that naming odors is the ultimate goal in odor memory studies (Lehrner et al., 1999), have long dominated the field of odor memory and contributed much to the distinction between the two forms of memory, but have often neglected to study veridical odor memory itself. One of the most recent and extreme examples is a study by Cessna and Frank (2013) in which they tried to answer the question whether odor knowledge or an odor naming strategy mediates the relationship between odor naming and recognition memory. Although this question may be of academic interest and the methods used to provide an answer to it were ingenious, one may wonder about their relevance for everyday life where we almost never name odors and the odors that are most important to us (odors of our surroundings and of people we know) are usually non-nameable. The fascination for identification as the way to do “object memory” research in the same way as in visual and verbal memory studies has in a way estranged the researchers of their subject. The few nameable odors in our life are almost certainly the ones that have lost their intimate relationship with places and situations and although to many authors nameable odors seem to be also ecologically most relevant, according to the MITSCOP they are much less interesting than the non-nameable odors that surround us but are not consciously remarked because they fit our expectations in the situation. Such odors are seldom used in odor memory experiments. The exceptions are collected air samples from odor polluted areas or from sick buildings, but these are usually only used to determine their detection thresholds and to characterize their intensity. To study veridical odor memory, it might be interesting to present subjects with the odors collected from rooms in their house and to check how well they could localize them. The nearest attempt to do something like this was that of Balez (2001) in France, who collected stories about the odors emanating from the different places in a shopping mall and about how regular visitors of the mall felt they could orient themselves and knew their position in the mall on the basis of them. Unfortunately, she did not verify their actual memory based orientation by presenting the odors to them and asking questions about their imagined position in the mall. It would have been a better proof of the way people use odor in their orientation than the highly artificial, but otherwise interesting and amusing experiment on scent-tracking by human subjects (Porter et al., 2005, 2007). They showed that people could follow a chocolate oil odor trail and that they probably used the lateral differences in odor intensity between the two nostrils. This reopened the old debate about the localization of odorant sources by birhinal differences in olfactory (Von Skramlik, 1924; Von Békésy, 1964) or in trigeminal (Kobal et al., 1989) stimulation. It was argued that active sniffing versus passive stimulation might play an important role in the question. Kobal et al., using passive sniffing, claimed that only odorants that also stimulated the trigeminal nerve showed localization and that purely olfactory stimulation did not. Stimulus concentration (Cometto-Muniz and Cain, 1990; Hummel et al., 2003; Frasnelli and Hummel, 2005; Frasnelli et al., 2009) or overall stimulus mass concentration (Cometto-Muniz and Cain, 1984) and/or the influence of stimulus volume (Frasnelli et al., 2011) were also indicated as possible factors. According to these authors, the role of active versus passive smelling in localization depended on the odorants used. Thus, mixed olfacto-trigeminal stimulants were better localized under passive conditions, but a pure odorant was better localized under active sniffing, probably due to increased olfactory attention as suggested by Zelano et al. (2005). In this connection it should be remarked that all experiments (both passive and active) were carried out under explicit perceptual conditions, but that there is of course a definite intentional difference between active sniffing and waiting for a stimulus to come. If one thinks about the difference between touching and being touched, one can easily imagine that in the case of olfaction the difference might also be important even in explicit experimental conditions. Of course there is also a large difference between the attention given to the stimulus in explicit laboratory experiments and the implicit and often unnoticed encounters with odors in everyday life. After all sniffing is usually limited to the few situations in which unexpected odors or new surroundings have to be inspected. In this respect almost all laboratory experiments are atypical for normal olfactory behavior and it will demand quite drastic steps on a number of aspects to bring the two closer together in order to provide ecologically valid insight in the role odor perception and memory play in our life.

## DO’S AND DON’TS IN ECOLOGICALLY VALID ODOR MEMORY RESEARCH

Generally speaking ecologically valid memory research should be based on incidental learning in an everyday situation and on implicit memory measurement. Apart from the earlier mentioned experiments by Degel and Köster (1999), Degel et al. (2001), only a few recent experiments meet these demands. Holland et al. (2005) showed the influence of incidental smelling of cleaning spirit on cleaning behavior and research in waiting rooms of Dutch hospitals showed that unnoticed odors can reduce aggressiveness and promote the perception of friendliness (LEV Report, 2012). The experiments on incidentally learned food memory did not respect the demand of implicit memory measurement. They asked people explicitly to recognize the food they had eaten under everyday circumstances and without any special attention and had people compare their memory of the food with new samples of the same food and slight variations of it. Although this type of measurement is not implicit, it provides much information about the implicit memory for incidentally learned food impressions. Thus, it has been shown, that the memory for sweetness and fattiness, may be distorted in some products whereas for other sensory aspects it remains intact (Mojet and Köster, 2002, 2005; Köster et al., 2004). Especially the method of relative memory measurement, in which people are asked to tell whether the presented samples are more, less or equally strong compared to the earlier incidentally eaten food, provides much information about changes in appreciation and perception of the food occurring in memory. It is difficult to obtain such information with purely implicit memory methods and responses to questions like “Is this product now worse or better liked than it was?” may nevertheless tell much about the way in which the memory was implicitly retained (e.g., whether the memory of the sweetness has faded, while the memory of the bitterness did not).

The more implicit ways of testing memory such as measuring preference and/or decision time in free choice among a set of alternatives after previous incidental exposure to one of them, or registering behavioral and facial changes to incidental stimuli (Fedoroff et al., 1997, 2003; Holland et al., 2005; Papies and Hamstra, 2010; Soussignan et al., 2012; Gaillet et al., 2013) often fail to provide such more detailed information. Thus, in order to do ecologically relevant memory research, it is perhaps more important to make sure that learning is truly incidental or takes place in the same way as in everyday life than to comply with the rule of implicit memory that no explicit link may be made with the learning event. In the case of Gaillet et al. (2013), who exposed people, who were waiting to take part in a meal, to afaint and unnoticed fruit odor, variation of that odor was used to show the specificity of the reaction. Melon odor led to the choice of more vegetable rich appetizers, whereas pear odor changed the dessert choices toward fruits and away from rich and fatty items. Such category specific reactions provide interesting and truly ecological information, but do not provide insight in memory distortions of the food itself as explicit relative memory measurements would. Of course, it is preferable to have an implicit memory measure first before asking explicit questions. Since subjects who have been exposed to explicit questions have lost their naivety and cannot be used again in incidental learning and implicit memory experiments, one should limit the use of such questions to the moment one is sure not to need the subject again. This limits the experimental possibilities. Thus, it is only possible to do within-subject research if the subject was incidentally exposed to different stimuli in the same session or in comparable sessions before the memory was tested, even with implicit methods (e.g., reaction time measurements). It is also important to avoid methods that imply identification of the stimuli either by name or otherwise and that objectify the odor in some form. As shown above in the experiments by Degel and Köster (1999), Degel et al. (2001), odors that can be identified by name, become “things” and tend to lose their intimate connections with the situations in which they occurred in a person’s life and therewith their main function. Odors are probably not meant to be identified. They are the silent emotional reminders of the surroundings and situations with which they are linked by unconscious association and they are powerful evokers of the feelings that belonged to these events. In fact, we have stored many thousands odors in that associative and unconscious way and we have perhaps only names for at most 50 of them (Schab, 1991; Sulmont et al., 2002). Usually, we even determine the name of the odor and its source by remembering first the situation in which we earlier encountered the odor (why does this odor make me think of the attic in the house of my grand-parents when I was looking for fishing gear? Ah, there were apples drying. It is the odor of drying apples!). Objectifying odors into objects is denying them an essential part of their function in life and although it may be useful in the study of olfactory perception mechanisms and in the industrial application of chemicals in the perfume industry, it destroys the possibility of studying their normal function in human life. The proponents of odor-object theories overlook this in their search for odor identification and discrimination as the end goal of all odor research. If odors are indeed not meant to be identified, but should, as stated in the misfit theory, be recognized as the ephemeral and unnoticed providers of feelings of safety and comfort, unless they are unknown and unexpected or out of place, we may need to devote more time to emotional effects of odor associations and to the investigation of incidentally learned situational odor memories instead of investigating how “odor objects” are constructed and changed by odor-odor and odor-taste learning under laboratory conditions with odors from bottles or olfactometers.

If indeed MITSCOP is right, situational experience with an odor will reduce the conscious perception of that odor upon repetition in the same surroundings or foods, but may remain unchanged or might even be enhanced in other environments or eating situations. The consequence for research is fundamental. It means that single measurements of the emotional effects of odors are not predictive of the longer term odor effects and that these effects are not odor-object dependent, as is often assumed, but are strongly linked to associations and depend on situational congruence. Perhaps only in artificial laboratory situations where the odors are presented explicitly as particular items alone or in combination with other odor or taste items as in the experiments on odor-odor learning or odor-taste learning (Stevenson, 2001a,b,c; Stevenson and Boakes, 2003) can the influence of situational effects be excluded or at least controlled. The external validity of that type of research could be questioned however. Within the limitations of the laboratory situation, the odors or flavors of the other items are the only situational context elements available and odors are therefore almost inevitably associated with them. Nevertheless, the same mechanisms seem to function in the real world as is illustrated by cross-cultural studies, which show that different cultural settings not only lead to differences in preference for flavors, but also to genuine differences in perception and discrimination (Ayabe-Kanamura et al., 1998).

In this respect the research by Baeyens et al. (1996) is perhaps most revealing. They incidentally exposed people to scented toilets in one experiment and to scented massage oil in another and showed that the liking for the odor was strongly dependent on the situational appreciation of the subjects, irrespective of whether they had consciously noted the odors during the exposure or not. Rozin et al. (1998) on the other hand tried to repeat this type of evaluative conditioning in a laboratory setting and had very little success. They ascribed their lack of success mainly to the laboratory surroundings and to the fact that the neutral odors they used might be particularly resistant to picking up emotional associations. The use of squeeze bottles for the explicit presentation of the odors and the use of very well-known odors (related to many different previous situations) may also have contributed to their failure.

**Table 2** presents a summary of the do’s and don’ts in performing ecologically relevant odor memory research.

**Table 2:** Overview of recommendations in ecologically valid odor memory research.

Feature	Do	Don’t	Comment (see also text below)
Learning exposure	Present stimuli incidentally in a natural situation	Draw attention to the target stimulus in any way	Avoid memory references
Subject choice	Select people that are naïve to memory experiments	Use the same people again after a memory test	Ask secrecy of your subjects
Stimulus selection	Choose situationally relevant stimuli	Choose very well-known and /or nameable stimuli	Provide imagined situational link
Stimulus presentation	Present naturally or at unnoticed strength	Present from odor bottles or olfactometers	Pre-tests necessary
Memory verification (implicit)	Give priority to implicit measurements	Present test stimuli in a way different from learning	Prepare natural alternatives of same category
Memory verification (explicit)	Absolute and relative memory measurements	Fatigue subjects with long questionnaires and why’s?	Select attributes for relative memory tests
Data treatment	Look for segments in your subject population	Average without looking for behavior differences	Prior analysis of behavioral differences
Data analysis	Analyze your hits, misses, false alarms, correct rejections	Calculate composites (*d*′) without verifying hits, correct rejections	Hit rate as smaller, larger, or equal to chance? Verify!
Characterization of memory effects	Verify distortions in relative memory	Forget to check differences in rel. attribute memory	Check memory mode: recollection or change detection
Repeated exposure effects	Use different subject groups. Vary amount of learning exposure	Expect that more exposure will have no effect on both liking and perception	Check influence of perceived complexity

The first four of these recommendations have been amply discussed above, but some of the comments in the table might need more clarification. Thus, avoiding memory references means that people should not be aware of participating in a memory experiment and that all allusions to memory should be avoided [see discussion on Olsson and Cain (2003) above]. Furthermore it is important to ask the people, who, after finishing the experiment, know that it was about memory, not to divulge this knowledge to others and to corroborate this demand by explaining that there is a prize for the person who has the best memory results and that telling others would reduce their chances of winning it. Providing a representative situational link by making people think of a situation in their life (either by providing images or telling them a story) may help to verify the influence of the appropriateness of the stimulus in this situation on the odor memory. Of course letting them participate in a real situation is preferable but imagination can work well especially via stories in which the subjects can imagine the situation in their own familiar surroundings.

Incidental learning in natural situations as in the Baeyens et al. experiments or by presenting the odors in a perceptible, but not spontaneously noted, way as in the experiments of Degel and Köster (1999), Degel et al. (2001), Fedoroff et al. (1997, 2003), Holland et al. (2005) and of Gaillet et al. (2013) is perhaps the best way to assess ecological validity of the results. Other forms of stimulus exposure (even in the laboratory from bottles or an olfactometer) may also be used as long as they are so well disguised as part of another research subject, that even the thought of them being used as memory targets does not arise in the experimental subjects. Thus, asking people in a laboratory setting to judge odors or flavors on their pleasantness or intensity and later presenting some of these odors in a relative memory test among slightly modified variations of them as distractors, might still provide ecologically valid information about the stability or distortion of the memory for them, even if the proper situational circumstances are not respected. The information obtained in this way is more limited however. If it relies on explicit memory verification as proposed here, this might perhaps be preceded or accompanied by implicit measurements such as face reading or psychophysiological measurements (heart rate, electro-dermal responses). If the emotions raised by the memory should be measured, it should be done before the explicit memory measurement and preferable in an implicit way (e.g., in a seemingly non-related projection test taken under the influence of the stimuli under the same unnoticed conditions). Once memory testing is made explicit, the emotional value of the stimulus will probably change and loose its ecological relevance.

## DATA TREATMENT

In treating the data, one should look for possible segments in the population that may differ in their behavior with regard to the stimuli involved or in the importance they attribute to them. Thus, it is known that with respect to eating chocolate the population is divided into two groups, those who bite and chew their chocolate and those who suck it, and the difference in the perception and memory of chocolate in these groups makes it difficult to make chocolate that is satisfying both groups. Averaging over such groups should therefore be avoided and prior segmentation on the basis of stimulus-related behavior is a prerequisite of good ecologically valid research. Experience with odors and flavors in a certain domain will also be an important criterion for segmenting. People who collect wines and keep them for aging and special occasions will appreciate and remember them differently than do wine novices.

In analyzing perceptual detection and memory data, Signal Detection Theory has played a predominant role over the last few decades and in many cases, the results are only presented in the form of the composite statistic *d*′ or similar measures. In the case of perceptual detection, where there is no doubt about what is the signal and what is noise, this use is obvious, but in incidentally learned odor memory where all signs point in the direction of novelty and change detection rather than recollection of the earlier encountered stimulus (Morin-Audebrand et al., 2012), the situation is less clear and in this case it is advisable to look at the components (hits, misses, false alarms, and correct rejections) as well as at the composite. Furthermore, it is worthwhile to compare the certainty of the subjects about their different statements. If indeed novelty prevails over recollection in this form of memory, correct rejection should be seen as the signal and it is not surprising that it is also the response the subjects are more certain of than of their hits, which they seem not to detect any better than by chance. Since this may truly reflect a mode of remembering (and one that fits well in the misfit theory), it seems important not to hide it under composites like d’. Verification whether the hit rate is better than chance and how sure the people are of their different responses is to be recommended as a first step in the verification of the form of the memory involved. As already described above, more detailed information about the memory effects can be obtained from relative memory measurements that involve comparison of the actual and remembered target.

That memory measurement necessarily involves a repeated encounter with the earlier learned stimulus may also have an influence on the memory content because repeated exposure to an odor may change the perceived quality of it, especially when the odor is new and complex (Köster and Mojet, 2007; Mojet and Köster, 2013). It may therefore be of interest to compare the memory for odors in a group that has been incidentally exposed only once to the odor with that of a group that has been exposed to it more (5–10) times. Especially when new odors are involved, it may well be that the memory of such a repetition group provides a more realistic image of the memorability of the odor when used more frequently in normal everyday life.

## RELEVANT ODOR MEMORY RESEARCH PARADIGMS

Three examples of ecologically relevant research paradigms are described, one devoted to pre-launch research for the introduction of a new flavor, one dedicated to possible uses as an environmental odor, and one directed at reduction of aggression or stimulation of pleasant behavior in public places. These examples stem from applied work that we have carried out and that have led to successful solutions. Here they are presented as suggestions for more relevant research. In our case they worked well, but much may depend on the circumstances and the people involved. In some of the cases described there was simply no funding and no time to do elaborate research comparing experimental and control groups. We hope that suggestions like these might stimulate readers to come out of their laboratories and to try some more ecologically relevant methods to answer real problems.

### ODOR OR FLAVOR MEMORY AS A PREDICTIVE ELEMENT IN PRE-LAUNCH RESEARCH

Suppose one had to choose between two alternative new formulas (A and B) for a product to be launched in an already existing market in which a competitor product (CP) is the market leader, how could flavor memory help in reaching the best decision? In answering this question we suppose that all traditional measures have been taken and that the three products A, B and CP (which serves as a benchmark) have been extensively described by a well-trained descriptive panel and have already been judged positively on a hedonic scale by a representative consumer panel or by different segments of the consumer population such as product users and non-users, or groups that differ in their use of the product (due to habit, age, etc.). In some cases, depending on the implicitness of the way the hedonic information was obtained, these groups could be used again for the memory testing. They might again be invited under a false pretense (e.g., an unrelated experiment) and then inadvertently be exposed to the three stimuli and a number of small variants of each of them in an absolute memory test in which they simply indicated whether they had recognized the one they had judged in the earlier session. After this they were confronted with a newly coded set of the same stimuli for a relative memory test in which they indicated whether the now presented stimuli were nicer, less nice or equally nice (and intense on a number of relevant attributes) as the similar shaped ones they had judged some time (a day, a week) ago. On the basis of the hit and correct rejection rates of the answers in the absolute memory, one could draw conclusions about the prevailing mode of the memory (recollection or change and novelty detection) and the relative memory would make it possible to see whether the product was more positively or negatively remembered and which of the significant attributes might have contributed to eventually found memory distortions. This provides important information for possible product improvements. In combination with some repeated presentation measurements (extended boredom test as described in (Köster and Mojet, 2007; Mojet and Köster, 2013), the comparison of the memory results for the new products with that of the benchmark in both user and non-user groups will provide better predictive information about the future market success of the new products than the simple first impression measurements on which most present pre-launch consumer research is based and may help reducing the risk of market flops considerably (see Köster and Mojet, 2012a,b).

### USE OF ODOR MEMORY IN THE IMPROVEMENT OF LIVING PLEASURE FOR MENTALLY HANDICAPPED OR VISUALLY HANDICAPPED PERSONS

In institutions for mentally or visually handicapped people, odors can be used quite effectively in several ways. 

#### Finding one’s way

In an institution for mentally handicapped persons in The Netherlands, a problem arose from the fact that several different corridors to the dormitory units had their access via a large hall and many of the inhabitants got lost trying to find their way home. Odorizing the different corridors with hardly detectable and spontaneously not noticed odors solved the problem. People “smelled home” at the corridor entrance and were hardly ever mistaken. In the same way odors have been used at corridor crossings in institutes for the visually handicapped. They learned very quickly what turn they should make.

#### Preparing for routines

Personnel working with mentally handicapped people often have difficulty obtaining their clients cooperation in the preparation for daily (meals) or regularly recurring events (going to the swimming pool). Hardly noticeable food odors or swimming pool odors have been used with success in making clients more cooperative by giving them an anticipatory pleasure that could not be matched by any other source of stimulation.

### REDUCING AGGRESSION AND STIMULATION OF PLEASANT BEHAVIOR IN PUBLIC PLACES

In emergency waiting rooms in hospitals where aggression and unfriendliness may arise easily from the fact that some later arrivals are treated more rapidly than others on account of their more acute need as judged by the staff, weak and not spontaneously noticed odors have been used with success to reduce aggressive behavior and stimulate friendliness between visitors and toward the personnel (LEV Report, 2012). The odors were chosen on the basis of a photographic projection test developed for judging the influence of unnoticed odors and of the presence of flowers on the appreciation of rooms and meals (Mojet, Holthuysen, Van Veggel, de Wijk and Köster, in preparation). The odors are also employed to try to reduce unpleasant behavior in public transport.

## FINAL CONCLUSIONS ON THE ROLE OF ODOR MEMORY IN EVERYDAY LIFE: MISFIT AND FIT

Odors guard our lives while not being noticed consciously most of the time. Thus they provide feelings of safety and comfort with the surroundings without demanding attention for themselves. They are not there to be named or identified, but to silently link us to the world and to our history of lived situations. When identified, odors lose this function. Most odors that fit our expectations remain unnoticed. Misfits are noted. Although it is also important, intentional smelling and the pleasures and displeasures it may provide is disproportionately overrepresented in olfactory research compared to its role in daily life. Applied research should be further developed, taking the special characteristics and functions of incidental odor memory into account.

## AUTHOR CONTRIBUTIONS

Egon P. Köster drafted, Per Møller and Jozina Mojet co-drafted the manuscript. All authors were involved in developing the first draft of the manuscript into the final version suitable for publication and they all approved the final manuscript.

## Conflict of Interest Statement

The authors declare that the research was conducted in the absence of any commercial or financial relationships that could be construed as a potential conflict of interest.
